# The association between *CD40* gene polymorphisms and the risk of cancer based on a meta-analysis

**DOI:** 10.3389/fgene.2026.1735761

**Published:** 2026-05-21

**Authors:** Sixin Li, Xichen Feng, Anjie Chen, Jiandong Gui, Chenwei Gu, Chen Guo, Yujie Deng, Yuanyuan Mi

**Affiliations:** 1 Department of Urology, Affiliated Hospital of Jiangnan University, Wuxi, Jiangsu, China; 2 Wuxi School of Medicine, Jiangnan University, Wuxi, Jiangsu, China

**Keywords:** biomarker, breast cancer, cancer susceptibility, CD40, polymorphism

## Abstract

**Background:**

CD40, a constituent of the tumor necrosis factor (TNF) receptor superfamily, exhibits variable expression across different cancer types. It plays a role in mediating tumor cell proliferation, apoptosis, and survival, as well as antitumor immune responses and the tumor microenvironment. Although some studies have suggested a potential association between *CD40* gene polymorphisms and cancer risk, definitive conclusions remain elusive.

**Methods:**

We conducted a comprehensive literature search across PubMed, Web of Science, Google Scholar, Embase, and relevant Chinese databases for studies published up to 3 February 2025. Our systematic analysis focused on elucidating the association between CD40 polymorphisms and cancer susceptibility, employing various comparative models and subgroup analyses. We analyzed the differential expression of CD40 between various tumor tissues and their corresponding normal tissues, as well as the impact of CD40 expression levels on the overall survival outcomes of cancer patients using the Gene Expression Profiling Interactive Analysis (GEPIA) database. Based on the NCBI database, we further investigated the distribution characteristics of the mutant allele for four single nucleotide polymorphisms (SNPs) in the *CD40* gene across six major global populations. Additionally, we constructed a protein–protein interaction network for CD40 using the STRING database.

**Results:**

By analyzing 10 high-quality studies (comprising 20 case–control studies), we illustrate that the specific *CD40* gene polymorphism (rs1883832) has a significant impact on breast cancer susceptibility. However, no significant correlation was found between the other three CD40 polymorphisms (rs4810485, rs1800686, and rs3765459) and tumor susceptibility.

**Conclusion:**

Our study found a strong association between CD40 polymorphisms (rs1883832) and breast cancer risk. However, a limitation of this study is that it did not explore the potential application of CD40 in the early diagnosis of breast cancer, nor did it clarify its impact on patient prognosis or its feasibility as a biomarker. These critical issues will be key directions for future research.

## 1 Introduction

CD40, a critical component of the tumor necrosis factor (TNF) receptor superfamily, is extensively expressed on immune cells, including B cells, dendritic cells, and macrophages, as well as on certain non-immune cells, such as endothelial and epithelial cells. Its ligand, CD40L (CD154), is primarily expressed on activated T cells. The interaction between CD40 and CD40L plays a vital role in adaptive immunity, facilitating processes such as B-cell activation, antibody class switching, and T-cell-mediated immune responses. Furthermore, CD40 signaling is essential for the activation of antigen-presenting cells, which is crucial for initiating antitumor immune responses (Elgueta et al., 2009). Owing to its central role in immune regulation, CD40 has emerged as a significant focus in cancer immunotherapy research (Vonderheide, 2020).

CD40, a member of the TNF family, is extensively expressed in normal cells and demonstrates significant expression in a variety of malignant hematopoietic cells and solid tumors. Its expression profile spans a wide array of malignancies, including leukemia, lymphoma, bladder cancer, melanoma, breast cancer, lung cancer, and colon cancer (Vonderheide, 2007; Slobodova et al., 2011; Wu et al., 2008; Ishikawa et al., 2008; Cooke et al., 1999; Uckun et al., 1990; Matsumura et al., 2016). Research suggests that CD40 is involved in tumor initiation and progression through multiple molecular mechanisms. In endothelial cells, activation of the CD40 signaling pathway can inhibit apoptosis and promote proliferation, potentially accelerating tumor growth by facilitating angiogenesis (Deregibus et al., 2003; Flaxenburg et al., 2004; Melter et al., 2000). Furthermore, the interaction between CD40 and CD153 can induce the production of chemokines such as IL-10 and VEGF in various cell types, which are crucial for the metastasis of malignant tumors (Murugaiyan et al., 2007). Importantly, the expression level of CD40 is positively correlated with the risk of angiogenesis in certain tumors (Huang et al., 2011), underscoring its critical role in tumor progression.

Despite its tumor-promoting properties, CD40 is increasingly recognized for its potential in antitumor therapy. The role of CD40 in cancer has been extensively investigated due to its ability to modulate antitumor immunity. Activating CD40 on dendritic cells enhances their capacity to present tumor antigens and stimulate cytotoxic T cells, leading to tumor regression (Beatty et al., 2011). It has been identified as a significant biomarker for various malignancies, including ovarian cancer (Wang et al., 2019) and hepatocellular carcinoma (Lorente, 2018). Research indicates that activation of the CD40 signaling pathway can markedly enhance immune responses within the tumor microenvironment, positioning CD40 as a promising target in cancer immunotherapy. Consequently, CD40 agonist therapies are currently undergoing evaluation in clinical trials for the treatment of multiple malignancies, such as non-Hodgkin’s lymphoma, advanced urothelial carcinoma, malignant melanoma, pancreatic ductal adenocarcinoma, renal cell carcinoma, non-small-cell lung cancer, and glioma (Piechutta and Berghoff, 2019). Notably, preclinical studies have shown that recombinant CD40 ligand therapy exerts significant antitumor effects in CD40-positive ovarian tumor models, while CD40 antagonists can augment the efficacy of cisplatin chemotherapy (Ghamande et al., 2001). These findings underscore the diagnostic and therapeutic significance of CD40 in oncology (Beatty et al., 2017).

CD40 is integral to tumor development and progression. Concurrently, research has demonstrated that *CD40* gene polymorphisms are significant genetic determinants not only of cancer susceptibility but also of the risk for various infectious and autoimmune diseases, including Graves’ disease (Jacobson et al., 2005) and coronary artery calcification (Jacobson et al., 2007). Recent studies have identified correlations between *CD40* gene polymorphisms and the incidence of multiple tumors, such as lung cancer, cervical cancer, breast cancer, and lymphoma. Nevertheless, the limited sample sizes of individual studies pose challenges in fully elucidating the precise relationship between CD40 polymorphisms and cancer risk. In addition, there have been no previous reports of meta-analysis on the association between CD40 polymorphisms and tumors. To address this, we systematically compiled data on the associations between four CD40 polymorphisms (rs1883832, rs4810485, rs1800686, and rs3765459) and various tumors.

## 2 Materials and methods

### 2.1 Bioinformatics analysis

The differential expression of CD40 across various tumor samples and paired normal tissues was examined using data from the Gene Expression Profiling Interactive Analysis (GEPIA) database. Moreover, the impact of varying CD40 expression on overall survival in certain malignant tumor patients was analyzed based on GEPIA data. The expression data of the target genes were retrieved from the GEPIA database. The expression level of each gene is represented in transcripts per million (TPM), a standardized unit for RNA-seq data that accounts for both gene length and sequencing depth, allowing for comparative analysis of gene expression across samples. In the figures generated from GEPIA, the Y-axis indicates the log_2_-transformed TPM value [log_2_ (TPM +1)] for better visualization of expression differences. For survival analyses, patients were stratified into “high-expression” and “low-expression” groups based on the median expression level of the target gene. The median expression value was employed as the cutoff threshold. Kaplan–Meier survival curves were then plotted to compare overall survival or disease-free survival between the two groups, and statistical significance was assessed using the log-rank test. The minor allele frequencies (MAFs) of the mutant allele for four SNPs of CD40 across six major global populations were analyzed using the 1000 Genomes browser (https://www.ncbi.nlm.nih.gov/snp). The CD40 interaction network is analyzed using the STRING database (version 12.0).

### 2.2 Data collection

A thorough literature review was executed using PubMed, Web of Science, Embase, and pertinent Chinese databases, with the latest search conducted on 3 February 2025. The search strategy incorporated keywords including “CD40,” “polymorphism,” “cancer,” “carcinoma,” and “tumor.” Additionally, the references of the selected studies were scrutinized to identify further relevant studies. Of 3,413 articles retrieved, only 10 articles met the inclusion criteria.

### 2.3 Inclusion and exclusion criteria

The studies included in the analysis were required to satisfy the following criteria: (a) examination of the association between cancer susceptibility and at least one of the four CD40 polymorphisms (rs1883832, rs4810485, rs1800686, and rs3765459); (b) employment of a case–control study design; and (c) provision of genotype data for both cases and controls or for specific genetic models. The exclusion criteria included (a) absence of a control group, which could impede the interpretation of results; (b) absence of genotype frequency data; and (c) duplicate publications, which were identified and excluded.

### 2.4 Data extraction for meta-analysis

The key variables extracted from the selected studies encompassed the authors’ names, year of publication, country of origin, ethnicity of participants, type of cancer, sample sizes for both cases and controls, source of the control group, assessment of Hardy–Weinberg equilibrium (HWE) in the control group, and the genotyping methods employed.

### 2.5 Data analysis

The relationship between four CD40 polymorphisms and cancer susceptibility was analyzed through the calculation of odds ratios (ORs) with 95% confidence intervals (CIs), based on genotype frequency comparisons between case and control groups. The statistical significance of the aggregated ORs was determined using the Z-test. Study heterogeneity was assessed using the chi-square-based Q-test; a p-value greater than 0.05 necessitated the application of a fixed-effects model, whereas a random-effects model was employed otherwise. The statistical analyses encompassed allelic contrast, homozygote comparison, dominant and recessive genetic models, and heterozygote comparison. The HWE in control groups was evaluated using the Pearson chi-squared test. Publication bias was examined using Begg’s and Egger’s tests. All statistical computations were conducted using Stata software (Version 11.0; StataCorp LP, College Station, TX).

## 3 Results

### 3.1 The association between CD40 and cancer: an analysis from GEPIA and NCBI databases

**FIGURE 1:**
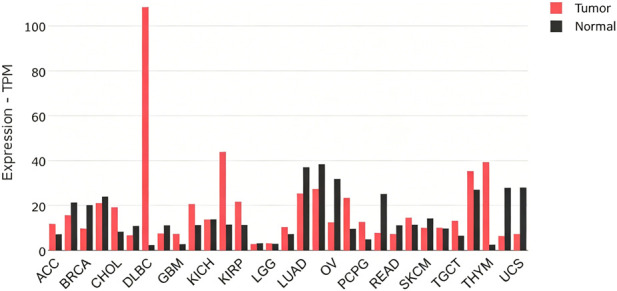
The CD40 expression profile was determined from the GEPIA database based on tumor samples and paired normal tissues, retrieved on 3 February 2025. The figure encompassed various cancer types, including adrenocortical carcinoma (ACC), breast invasive carcinoma (BRCA), cholangiocarcinoma (CHOL), lymphoid neoplasm diffuse large B-cell lymphoma (DLBC), glioblastoma multiforme (GBM), kidney chromophobe (KICH), kidney renal papillary cell carcinoma (KIRP), brain lower-grade glioma (LGG), lung adenocarcinoma (LUAD), ovarian serous cystadenocarcinoma (OV), pheochromocytoma and paraganglioma (PCPG), rectum adenocarcinoma (READ), skin cutaneous melanoma (SKCM), testicular germ cell tumors (TGCTs), thymoma (THYM), and uterine carcinosarcoma (UCS).

**FIGURE 2:**
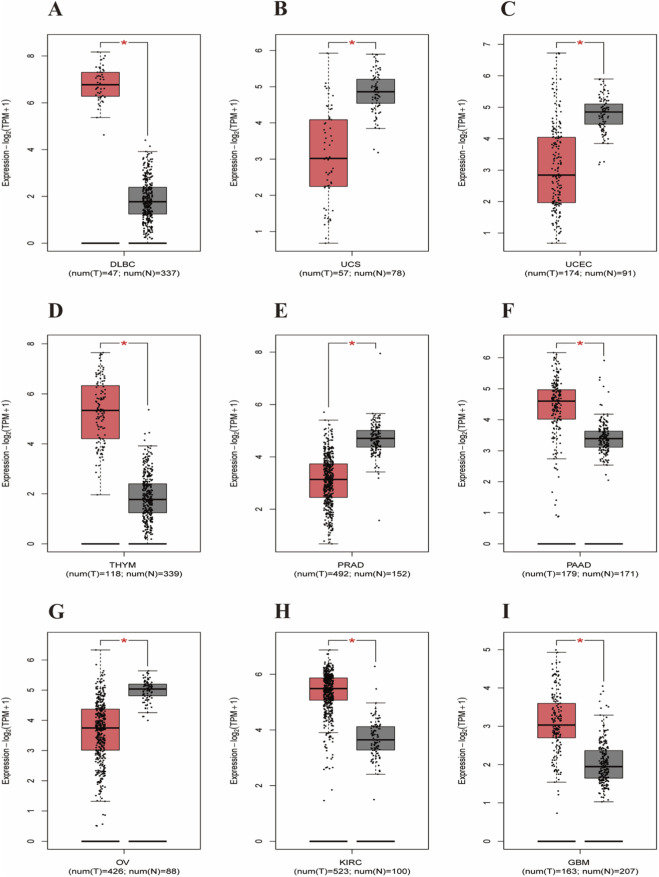
On 3 February 2025, GEPIA database analysis revealed significant differences in CD40 expression between nine tumor samples and their paired normal tissues. *CD40* gene expression is higher in diffuse large B-cell lymphoma (DLBC) tumor samples (n = 47) than in paired normal tissues (n = 337), with *P* ≤ 0.05 **(A)**. *CD40* gene expression is lower in uterine carcinosarcoma (UCS) tumor samples (n = 57) than in paired normal tissues (n = 78), with *P* ≤ 0.05 **(B)**. *CD40* gene expression is lower in uterine corpus endometrial carcinoma (UCEC) tumor samples (n = 174) than in paired normal tissues (n = 91), with *P* ≤ 0.05 **(C)**. *CD40* gene expression is higher in thymoma (THYM) tumor samples (n = 118) than in paired normal tissues (n = 339), with *P* ≤ 0.05 **(D)**. *CD40* gene expression is lower in prostatic adenocarcinoma (PRAD) tumor samples (n = 492) than in paired normal tissues (n = 152), with *P* ≤ 0.05 **(E)**. *CD40* gene expression is higher in pancreatic adenocarcinoma (PAAD) tumor samples (n = 179) than in paired normal tissues (n = 171), with *P* ≤ 0.05 **(F)**. *CD40* gene expression is lower in ovarian serous cystadenocarcinoma (OV) tumor samples (n = 426) than in paired normal tissues (n = 88), with *P* ≤ 0.05 **(G)**. *CD40* gene expression is higher in kidney renal clear cell carcinoma tumor samples (n = 523) than in paired normal tissues (n = 100), with *P* ≤ 0.05 **(H)**. *CD40* gene expression is higher in glioblastoma multiforme (GBM) tumor samples (n = 163) than in paired normal tissues (n = 207), with *P* ≤ 0.05 **(I)**.

**FIGURE 3:**
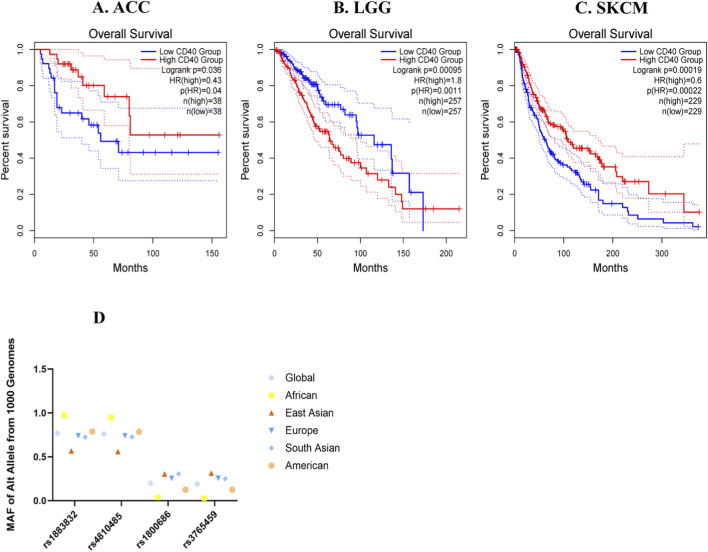
**(A)** In adrenocortical carcinoma (ACC), patients with high CD40 TPM have better OS than those with low CD40 TPM, number high = 38, number low = 38 [n (high) = 38, n (low) = 38], *P* ≤ 0.05. **(B)** In brain lower-grade glioma (LGG), patients with low CD40 TPM have better OS than those with high CD40 TPM, [n (high) = 257, n (low) = 257], *P* ≤ 0.05. **(C)** In skin cutaneous melanoma (SKCM), patients with high CD40 TPM have better OS than those with low CD40 TPM, [n (high) = 229, n (low) = 229], *P* ≤ 0.05. **(D)** MAF of the Alt allele (mutant allele) for the four SNPs from the online 1000 Genome.

The differential expression profile of CD40 between tumor samples of different cancer types and matched normal tissues was determined through preliminary analysis of the GEPIA database (Figure 1). It was found that the expression of CD40 was significantly different in nine tumors (Figure 2), which was related to the overall survival outcomes of adrenocortical carcinoma, brain lower-grade glioma, and skin cutaneous melanoma (Figures 3A–C). Subsequently, we analyzed the MAF of the mutant allele of four SNPs (rs1883832, rs4810485, rs1800686, and rs3765459) across six major global populations using the 1000 Genomes browser (Figure 3D).

### 3.2 Systematic review of the literature and selection of studies

**FIGURE 4:**
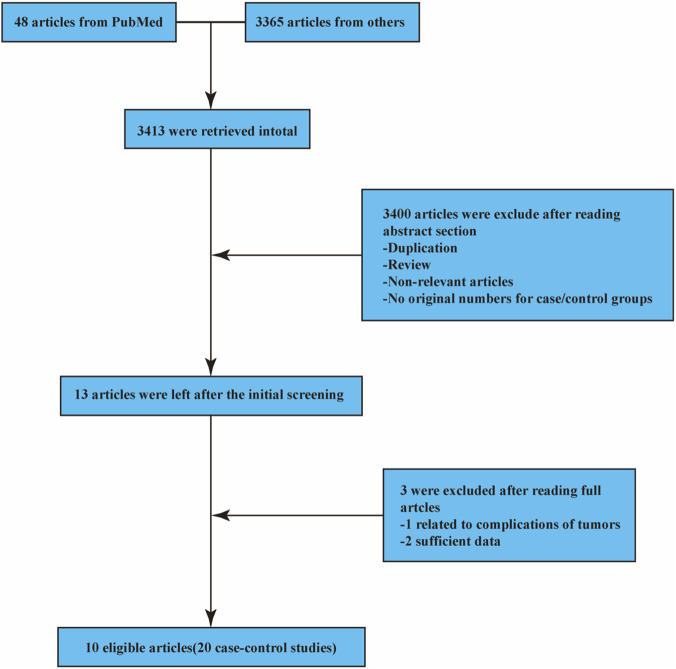
Flow chart depicting the search and screening strategies for the polymorphisms of CD40 (rs1883832, rs4810485, rs1800686, and rs3765459) studies from several databases.

Using an extensive database mining approach, we identified four functionally significant polymorphisms (rs1883832, rs4810485, rs1800686, and rs3765459) situated within the promoter region of the *CD40* gene. By systematically searching PubMed, Web of Science, Embase, and major Chinese databases with the latest search conducted on 3 February 2025, a total of 3,413 potentially relevant articles were obtained. After preliminary screening according to the pre-established inclusion criteria, 3,400 non-compliant papers were excluded. After full-text evaluation of the remaining 13 articles, one was excluded due to study and tumor complications, and two were excluded due to critical incompleteness that did not meet the inclusion criteria. Finally, 10 high-quality studies (comprising 20 case–control studies) that met all inclusion criteria were included in this meta-analysis (Nieters et al., 2011; Dimitrakopoulos et al., 2021; Zhou et al., 2015; He et al., 2022; Wang et al., 2017; Yan et al., 2017; Zhu et al., 2023; Krishnappa et al., 2017; Shuang et al., 2011; Dolen et al., 2010). The literature screening process is shown in Figure 4, and the basic characteristics of the included studies are summarized in Table 1. All information concerning the literature was presented, including first author, number of controls and cases, type of *CD40* gene polymorphisms, year of publication, ethnicity, genotyping method, and control sources (Table 1), and genotype counts of the analyzed polymorphisms of studies included in the meta-analysis are shown in Table 2.

**TABLE 1:** Characteristics of studies included in the meta-analysis.

Author	Year	Origin	Ethnicity	Cancer type	Source	Case	Control	Polymorphism	Method
Dimitrakopoulos	2021	Greek	Caucasian	Lung cancer	HB	228	299	rs1883832 C>T	Real-time PCR
Zhou	2015	China	Asian	Lung cancer	HB	105	109	rs1883832 C>T	PCR–RFLP
Wang	2017	China	Asian	Lung cancer	HB	467	384	rs1883832 C>T	TaqMan PCR
​	​	​	​	​	​	​	​	rs4810485 G>T	​
He	2022	China	Asian	Lung cancer	HB	400	400	rs1883832 C>T	MassARRAY
Nieters	2011	Germany	Caucasian	Non-Hodgkin’s lymphoma	HB	2613	3,600	rs1883832 C>T	Pyrosequencing and TaqMan PCR
Yan	2017	China	Asian	Cervical cancer	HB	227	483	rs1883832 C>T	TaqMan PCR
​	​	​	​	​	​	​	​	rs4810485 G>T	​
Chen	2011	China	Asian	Breast cancer	HB	591	600	rs1883832 C>T	PCR–RFLP
​	​	​	​	​	​	​	​	rs4810485 G>T	​
​	​	​	​	​	​	​	​	rs1800686 G>A	​
​	​	​	​	​	​	​	​	rs3765459 G>A	​
Dolen	2010	Turkey	Caucasian	Breast cancer	HB	145	94	rs1883832 C>T	PCR–RFLP
Krishnappa	2017	Malaysia	Asian	Cervical cancer	HB	70	61	rs1883832 C>T	PCR–RFLP
​	​	​	​	​	​	​	​	rs4810485 G>T	​
​	​	​	​	​	​	​	​	rs1800686 G>A	​
​	​	​	​	​	​	​	​	rs3765459 G>A	​
Zhu	2023	China	Asian	Cervical cancer	HB	421	504	rs4810485 G>T	PCR and next-generation sequencing
​	​	​	​	​	​	​	​	rs1800686 G>A	​
​	​	​	​	​	​	​	​	rs3765459 G>A	​

HB, hospital-based; SOC, source of control; PCR–RFLP, polymerase chain reaction followed by restriction fragment length polymorphism; PCR, polymerase chain reaction; MassARRAY, matrix-assisted laser desorption/ionization time-of-flight mass spectrometry.

**TABLE 2:** Genotype counts of the analyzed polymorphisms of studies included in the meta-analysis.

Polymorphism	Study	MM (cases)	WM (cases)	WW (cases)	W (%) (cases)	MM (controls)	WM (controls)	WW (controls)	W (%) (controls)	HWE
rs1883832	Dimitrakopoulos	54	61	113	287 (63%)	73	118	108	334 (56%)	<0.05
​	Zhou	19	50	36	122 (58%)	10	46	53	152 (70%)	0.9967
​	Wang	62	189	209	607 (66%)	56	184	139	462 (61%)	0.6986
​	He	98	182	120	422 (53%)	56	189	155	499 (62%)	0.8940
​	Nieters	229	1041	1343	3,727 (71%)	242	366	992	2350 (73%)	<0.05
​	Yan	36	103	84	271 (61%)	89	213	181	575 (60%)	0.0623
​	Chen	71	297	213	723 (62%)	69	241	274	789 (68%)	0.1563
​	Dolen	16	72	57	186 (64%)	10	45	39	123 (65%)	0.5728
​	Krishnappa	20	0	0	0 (0%)	4	0	0	0 (0%)	<0.05
rs4810485	Wang	63	186	208	602 (66%)	57	174	124	422 (59%)	0.7560
​	Yan	38	101	80	261 (60%)	89	212	180	572 (59%)	0.0599
​	Zhu	46	201	171	543 (65%)	54	222	224	670 (67%)	0.9275
​	Chen	73	299	206	711 (62%)	74	252	274	800 (67%)	0.1779
​	Krishnappa	0	1	8	17 (94%)	0	0	5	10 (100%)	<0.05
rs1800686	Zhu	28	206	185	576 (69%)	56	225	220	665 (66%)	0.8931
​	Chen	81	234	265	764 (66%)	53	251	293	837 (70%)	0.9423
​	Krishnappa	6	7	4	15 (44%)	5	6	6	18 (53%)	0.2291
rs3765459	Zhu	26	210	182	574 (69%)	53	231	219	669 (67%)	0.4904
​	Chen	79	235	258	751 (66%)	52	247	286	819 (70%)	0.8982
​	Krishnappa	0	4	13	30 (88%)	0	2	4	10 (83%)	0.6242

W/M, wild-type/mutant; HWE, Hardy–Weinberg equilibrium.

### 3.3 Analysis of CD40 polymorphisms and cancer risk

**FIGURE 5:**
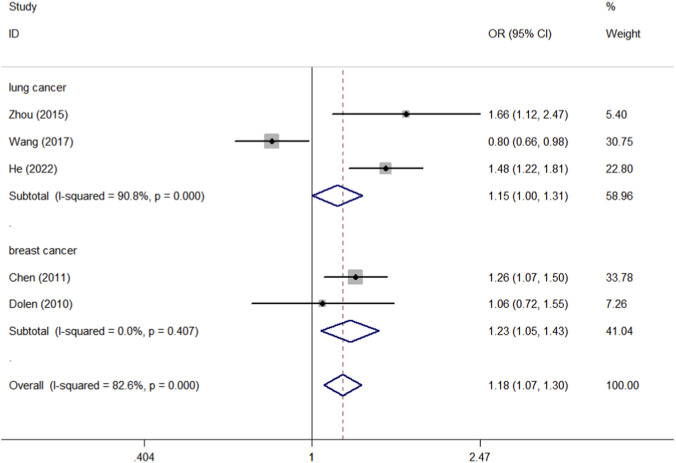
Forest plot of the association between the CD40 rs1883832 polymorphism and cancer susceptibility, stratified by cancer type. The meta-analysis was conducted under the allelic genetic model (T-allele vs. C-allele). The squares and horizontal lines correspond to the study-specific OR and 95% CI. The area of the squares reflects the weight (inverse of the variance). The diamond represents the summary OR and 95% CI.

**FIGURE 6:**
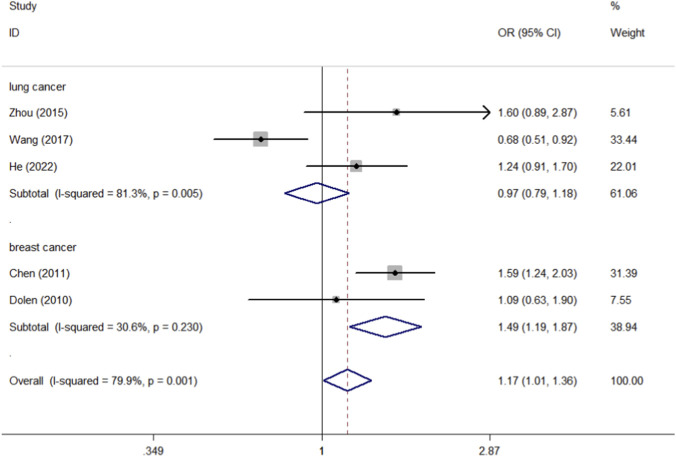
Forest plot of the association between the CD40 rs1883832 polymorphism and cancer susceptibility, stratified by cancer type. The meta-analysis was conducted under the allelic genetic model (CT-allele vs. CC-allele). The squares and horizontal lines correspond to the study-specific OR and 95% CI. The area of the squares reflects the weight (inverse of the variance). The diamond represents the summary OR and 95% CI.

**FIGURE 7:**
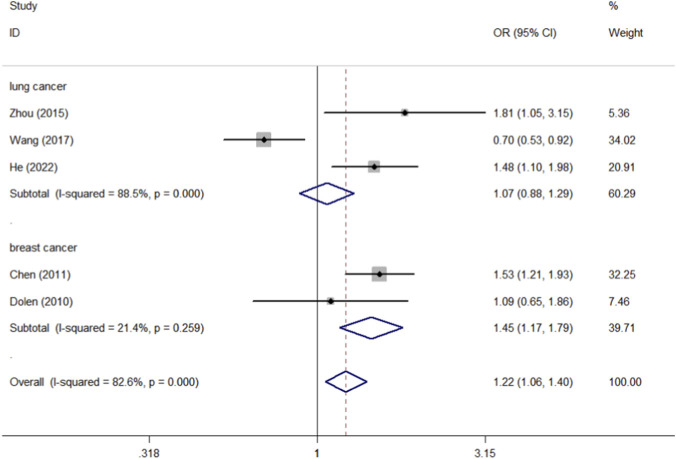
Forest plot of the association between the CD40 rs1883832 polymorphism and cancer susceptibility, stratified by cancer type. The meta-analysis was conducted under the allelic genetic model (TT + CT-allele vs. CC-allele). The squares and horizontal lines correspond to the study-specific OR and 95% CI. The area of the squares reflects the weight (inverse of the variance). The diamond represents the summary OR and 95% CI.

By collating the data of these 10 studies, we found that there were 4,799 cases and 5,952 controls of CD40 rs1883832, 1,681 cases and 1,941 controls of CD40 rs4810485, and 1,016 cases and 1,115 controls of CD40 rs1800686. CD40 rs3765459 consisted of 1,007 cases and 1,094 controls. The control group consists mainly of healthy individuals. We first analyzed the association between the four CD40 polymorphisms (rs1883832, rs4810485, rs1800686, and rs3765459) and overall cancer risk, followed by subgroup analysis by race and tumor type, all of which are found in Table 3. Meta-analysis of rs1883832 polymorphism demonstrated a significant association with cancer risk within the breast cancer subgroup (T-allele vs. C-allele, OR = 1.228, 95% CI = 1.050–1.435, *P*_heterogeneity_ = 0.407, P = 0.010; CT-allele vs. CC-allele, OR = 1.490, 95% CI = 1.190–1.866, *P*_heterogeneity_ = 0.230, P = 0.001; TT + CT-allele vs. CC-allele, OR = 1.446, 95% CI = 1.167–1.791, *P*_heterogeneity_ = 0.259, P = 0.001) (Figure 5, Figure 6, Figure 7), but no correlation was observed with the overall cancer risk. In contrast, other CD40 polymorphisms (rs4810485, rs1800686, and rs3765459) did not exhibit any significant correlations with cancer risk (Table 3).

**TABLE 3:** Stratified analysis of the association between *CD40* gene polymorphisms and cancer susceptibility after excluding studies with an HWE p-value <0.05.

Variable	No. of studies	Cases/controls	M-allele vs. W-allele [OR (95% CI), *P*_h_, *P*]	MM vs. WW [OR (95% CI), *P*_h_, *P*]	MW vs. WW [OR (95% CI), *P*_h_, *P*]	MM + MW vs. WW [OR (95% CI), *P*_h_, *P*]	MM vs. MW + WW [OR (95% CI), *P*_h_, *P*]
CD40 rs1883832							
Total	6	1,918/2,049	1.152 (0.923–1.439), 0.000, 0.212	1.298 (0.855–1.971), 0.001, 0.222	1.147 (0.854–1.539), 0.001, 0.362	1.189 (0.878–1.610), 0.000, 0.264	1.200 (0.860–1.675), 0.011, 0.283
Ethnicity							
Asian	5	1,773/1,955	1.169 (0.909–1.503), 0.000, 0.224	1.331 (0.834–2.125), 0.001, 0.230	1.156 (0.827–1.616), 0.000, 0.396	1.206 (0.854–1.702), 0.000, 0.287	1.223 (0.843–1.774), 0.005, 0.289
Cancer type							
Lung cancer	3	965/888	1.238 (0.772–1.983), 0.000, 0.376	1.605 (0.672–3.829), 0.000, 0.287	1.067 (0.647–1.759), 0.005, 0.799	1.199 (0.659–2.179), 0.000, 0.552	1.515 (0.822–2.791), 0.008, 0.183
Breast cancer	2	726/678	1.228 (1.050–1.435), 0.407, 0.010	1.286 (0.909–1.819), 0.700, 0.156	1.490 (1.190–1.866), 0.230, 0.001	1.446 (1.167–1.791), 0.259, 0.001	1.039 (0.751–1.439), 0.995, 0.816
CD40 rs4810485							
Total	4	1,672/1936	1.013 (0.817–1.257), 0.003, 0.904	1.001 (0.812–1.234), 0.107, 0.993	1.070 (0.728–1.572), 0.000, 0.731	1.050 (0.728–1.514), 0.000, 0.795	0.953 (0.784–1.157), 0.863, 0.625
Cancer type							
Cervical cancer	2	637/981	1.052 (0.907–1.221), 0.529, 0.499	1.039 (0.755–1.429), 0.646, 0.815	1.142 (0.918–1.419), 0.659, 0.233	1.119 (0.911–1.375), 0.576, 0.285	0.972 (0.724–1.305), 0.742, 0.849
CD40 rs1800686							
Total	3	1,016/1115	1.070 (0.941–1.216), 0.064, 0.305	1.116 (0.469–2.654), 0.004, 0.804	1.063 (0.887–1.272), 0.805, 0.510	1.083 (0.912–1.284), 0.578, 0.363	1.043 (0.442–2.461), 0.002, 0.923
Cancer type							
Cervical cancer	2	436/518	0.915 (0.755–1.108), 0.353, 0.363	0.646 (0.403–1.036), 0.229, 0.070	1.102 (0.843–1.442), 0.583, 0.477	1.008 (0.779–1.304), 0.453, 0.953	0.616 (0.394–0.963), 0.282, 0.034
CD40 rs3765459							
Total	3	1,007/1094	1.068 (0.938–1.216), 0.074, 0.324	1.011 (0.362–2.823), 0.001, 0.984	1.068 (0.891–1.280), 0.851, 0.479	1.085 (0.913–1.289), 0.598, 0.355	0.974 (0.341–2.779), 0.001, 0.960

*P*_h_, value of *Q*-test for the heterogeneity test; *P*, *Z*-test for the statistical significance of the OR; W/M, wild-type/mutant.

### 3.4 CD40 interaction network analysis

**FIGURE 8:**
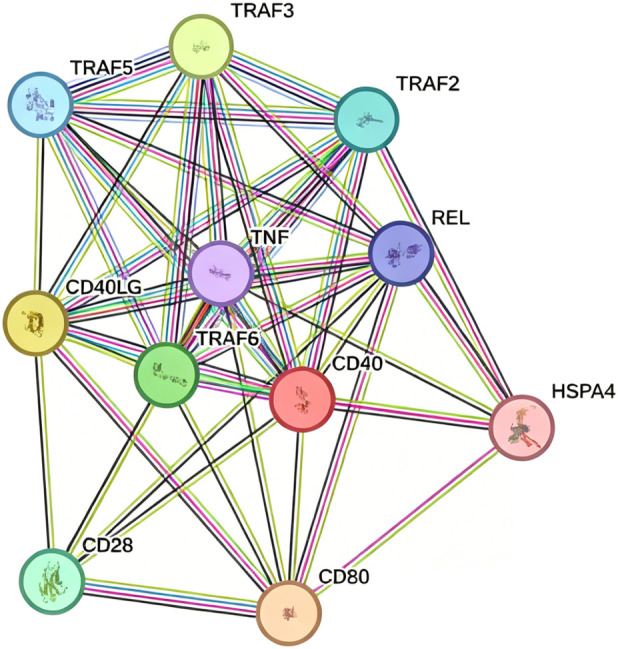
CD40 interacts with 10 predicted proteins from the STRING online website (version 12.0), acquired on 3 February 2025. CD80, CD40LG, TNF receptor-associated factor 3 (TRAF3), TNF receptor-associated factor 6 (TRAF6), CD28, TNF receptor-associated factor 2 (TRAF2), TNF receptor-associated factor 5 (TRAF5), proto-oncogene c-Rel (REL), tumor necrosis factor (TNF), and heat shock protein family A member 4 (HSPA4).

The analysis conducted using the STRING database revealed 10 potential genes that interact with CD40, thereby establishing a complex regulatory network (Figure 8). The genes identified in interaction with CD40 include CD80, CD40LG, TNF receptor-associated factor 3 (TRAF3), TNF receptor-associated factor 6 (TRAF6), CD28, TNF receptor-associated factor 2 (TRAF2), TNF receptor-associated factor 5 (TRAF5), proto-oncogene c-Rel (REL), TNF, and heat shock protein family A member 4 (HSPA4). These interactions indicate potential applications for combinatorial biomarkers in the early detection of cancer and offer valuable insights for future mechanistic investigations.

## 4 Discussion

To further explore the relationship between *CD40* gene polymorphisms and cancer susceptibility, we performed a meta-analysis comprising 18,605 participants, which included 8,503 cancer cases and 10,102 healthy controls. This study constitutes the first systematic assessment of the association between the *CD40* gene polymorphisms (rs1883832, rs4810485, rs1800686, and rs3765459) and the cancer risk. Our findings indicate that the mutant genotype at the CD40 rs1883832 is significantly correlated with an elevated risk of breast cancer, whereas no significant associations were observed for the other polymorphisms (rs4810485, rs1800686, and rs3765459). These results provide novel insights into the critical role of *CD40* gene polymorphisms in the etiology and progression of breast cancer.

Genetic polymorphisms are intricately linked to the onset, progression, and therapeutic response of cancer. Genetic polymorphism denotes the presence of two or more alleles at a particular gene locus within a population. These genetic variations can affect gene function or expression, consequently modifying an individual’s susceptibility to cancer, influencing the biological behavior of tumors, and impacting their response to treatment. For instance, polymorphisms within the matrix metalloproteinase gene family are significantly associated with tumor invasion and metastasis (Egeblad and Werb, 2002), whereas polymorphisms in the human leukocyte antigen gene are correlated with immune evasion and the efficacy of immunotherapy in cancer (Chowell et al., 2018).

Breast cancer represents one of the most prevalent malignant neoplasms affecting women globally, with its incidence increasing annually (Nounu et al., 2022). CD40 is implicated in tumorigenesis and tumor progression as studies have demonstrated its involvement in oncogenic processes through mechanisms such as the inhibition of tumor cell apoptosis (Deregibus et al., 2003; Flaxenburg et al., 2004; Melter et al., 2000) and the promotion of tumor angiogenesis (Murugaiyan et al., 2007). Although polymorphisms in the *CD40* gene have been associated with an increased risk of breast cancer, the precise mechanisms underlying this association remain to be elucidated. Of particular concern is triple-negative breast cancer (TNBC), the most aggressive breast cancer subtype, which constitutes 15%–20% of breast cancer cases (Arroyo-Crespo et al., 2019; Kim et al., 2018). This subtype is characterized by the absence of HER2 amplification and hormone receptor expression, presenting significant challenges for clinical treatment. However, due to the paucity of research data, definitive conclusions regarding the relationship between CD40 polymorphisms and TNBC risk have yet to be established. Moreover, CD40 has shown considerable promise in antitumor therapy. Research has demonstrated that it not only induces tumor regression (Beatty et al., 2011) but also functions as a biomarker for ovarian cancer (Wang et al., 2019) and hepatocellular carcinoma (Lorente, 2018) while augmenting immune responses within the tumor microenvironment. Of particular significance is the discovery that CD40 can substantially enhance the efficacy of cisplatin in ovarian cancer chemotherapy (Ghamande et al., 2001), offering novel insights for the clinical application of CD40. At the physiological and genetic levels, Mendelian randomization studies indicate that elevated CD40 levels are causally associated with an increased risk of estrogen receptor-positive (ER+) breast cancer, suggesting that sustained CD40 signaling may promote tumorigenesis by driving a chronic inflammatory microenvironment (Yi et al., 2025). In the context of therapeutic intervention, agonistic CD40 antibody (aCD40) therapy demonstrates significant antitumor potential. Its core mechanism involves targeting and activating key antigen-presenting cells of the innate immune system—particularly type 1 conventional dendritic cells (cDC1)—promoting their maturation, upregulating costimulatory molecules (e.g., CD80/CD86), and enhancing their ability to cross-present tumor antigens. This effectively bridges innate and adaptive immunity, initiating a robust CD8^+^ T-cell response (Lam et al., 2026; Chaib et al., 2022). Furthermore, aCD40 can remodel the immunosuppressive tumor microenvironment; for example, in combination with PKC agonists, it suppresses the expansion of myeloid-derived suppressor cells (MDSCs) and promotes their differentiation toward an immunostimulatory phenotype while also driving the infiltration of cytotoxic T cells into the tumor core (Chaib et al., 2022). Preclinical models confirm that aCD40 monotherapy or its combination with immune checkpoint inhibitors not only induces T-cell-dependent tumor regression but also establishes long-term immune memory, highlighting its pivotal role in modulating innate immunity to elicit durable antitumor responses (Lam et al., 2026). Nevertheless, it remains unclear whether *CD40* genetic polymorphisms influence breast cancer through innate immune mechanisms, specifically whether the CD40 rs1883832 Kozak sequence variant may affect breast cancer risk by modulating translation efficiency and downstream immune signaling pathways. Although we identified a significant association between the CD40 rs1883832 mutant genotype and breast cancer risk, limitations such as the scarcity of the relevant literature and the lack of individual-level data—which precludes adjustment for key residual confounders (e.g., age, environmental exposures, and hormonal levels)—must be acknowledged. Therefore, we can only conclude that the CD40 rs1883832 mutant genotype is associated with an increased risk of breast cancer development.

Through an analysis of the STRING database, we identified that CD40 interacts with proteins such as CD80, CD40LG, TRAF3, TRAF6, CD28, TRAF2, TRAF5, REL, TNF, and HSPA4 (Figure 7). Protein–protein interactions are integral to biological processes, influencing nearly all cellular activities, including cell signaling, metabolic regulation, gene expression, immune responses, disease progression, and drug discovery (Pawson and Nash, 2003; Ideker and Sharan, 2008; Ryan and Matthews, 2005). Research has demonstrated that tumor cells can manipulate signaling pathways, the cell cycle, apoptosis, metabolism, and immune evasion through aberrant protein interactions, thereby facilitating their survival, proliferation, and metastasis. These interaction data provide valuable insights for further investigation into the role of CD40 in tumorigenic mechanisms.

This study, however, is subject to certain limitations. First, the limited data available from the included studies and the inability to conduct subgroup meta-analyses for CD40 rs3765459 necessitate cautious interpretation of the results. Second, some studies did not strictly adhere to the standards for healthy control groups, and the heterogeneity of confounding factors across studies may introduce bias. Lastly, cancer is the result of the interplay of multiple factors, such as age, menstrual history, family history, and dietary habits in the case of breast cancer, which were not incorporated into this analysis—a common limitation in meta-analyses of gene polymorphisms and cancer. Despite these limitations, this review provides a foundation for enhancing breast cancer prevention, precise diagnosis, and innovative personalized treatment strategies by advancing scientific progress. Further in-depth and systematic research is necessary to gain a more comprehensive understanding of the impact of CD40 polymorphisms on breast cancer.

## 5 Conclusion

This comprehensive meta-analysis demonstrates a significant association between the CD40 rs1883832 polymorphism and breast cancer susceptibility. Although this finding strengthens the genetic link between CD40 and breast cancer risk, the clinical implications regarding its diagnostic, prognostic, or therapeutic utility remain to be elucidated. Future studies are warranted to investigate the potential of CD40, particularly functional polymorphisms such as rs1883832, as a biomarker for genetic predisposition to breast cancer, which may inform personalized risk assessment and preventive strategies upon further validation.

## Data Availability

The original contributions presented in the study are included in the article/supplementary material; further inquiries can be directed to the corresponding author.
